# Health Functionalities of Betaine in Patients With Homocystinuria

**DOI:** 10.3389/fnut.2021.690359

**Published:** 2021-09-09

**Authors:** Chelsea Truitt, Wouter D. Hoff, Ratnakar Deole

**Affiliations:** ^1^Department of Biochemistry and Microbiology, Oklahoma State University-Center for Health Sciences, Tulsa, OK, United States; ^2^Department of Microbiology and Molecular Genetics, Oklahoma State University, Stillwater, OK, United States

**Keywords:** homocystinuria, cystathionine-β-synthase, homocysteine, betaine, metabolism

## Abstract

Homocystinuria is a medical condition that can have widespread and harmful effects on multiple organ systems within the body. This disease is caused by a deficiency in one of the enzymes involved in the methionine metabolism pathway. One example would be a deficiency in cystathionine-β-synthase (CBS), which is seen in classical homocystinuria. A deficiency in CBS can lead to elevated levels of homocysteine (HCY) and possible depletion of methionine and/or cysteine. There are several different treatment options for patients with this condition, one of which is the administration of the drug betaine. Here we review the use of betaine to decrease these elevated levels of homocysteine back to within normal ranges. Published literature indicates that the use of this choline derivative is most beneficial to patients who are either not compliant with the recommended low methionine and low protein diet or wish to consume a less restricted diet.

## Introduction

Homocystinuria is a genetically inherited metabolic disorder that can have varying disease origins. Some patients for example, have a deficiency in the enzyme 5,10-methylenetetrahydrofolate reductase (MTHFR) (1). This results in the inability to produce the methyl donor 5-methyltetrahydrofolate and thus the remethylation reaction that normally occurs to convert the intermediate homocysteine back to methionine cannot take place. Another form of homocystinuria involves a deficiency of the enzyme cystathionine-β-synthase within the transsulfuration pathway (2). It is a genetic disorder with an autosomal recessive mode of inheritance involving a mutation or deficiency in the enzyme cystathionine-β-synthase. Genetic disorders that are autosomal recessive require the affected person to be homozygous and have both recessive alleles for that specific gene (aa). This means that both parents either had to be a heterozygous carrier (Aa) for that affected allele or they had to be homozygous and affected themselves (aa) (1).

Cystathionine-β-synthase converts the non-protein amino acid homocysteine to the next substrate within the pathway, cystathionine, which is then converted to cysteine via cystathionine-γ-lyase. A deficiency in cystathionine-β-synthase results in the accumulation of homocysteine within the body as well as a deficiency of cysteine (3). This accumulation most commonly has detrimental effects on the nervous, cardiovascular, skeletal, and ocular systems (4, 5). CBS deficiency is the most common form of homocystinuria and has a frequency range of 1:60,000 to 1:300,000 live births. While it is a rare disorder, it has detrimental effects on the affected patients. The available treatments for CBS deficiency vary widely and are dependent on the patient's preferences and responsiveness to treatment. Some patients can be treated with a vitamin *B*_6_ supplement while others might benefit more from following a restricted diet or taking a medication such as betaine (1).

Betaine is a choline derivative that occurs naturally within cells of mammals as well as plants, normally in response to osmotic stress. It can also be consumed through our diet when ingesting certain vegetables and seafood (6). Betaine is known to have 3 main functions. First, as mentioned previously, it plays a role in helping cells maintain a normal cell volume. It also works as a “chemical chaperone” that helps protect proteins from being denatured. Finally, it works as a methyl donor to the intermediate homocysteine within the transsulfuration pathway. This final function is how this compound is used to treat elevated homocysteine levels in homocystinuria. Metabolism of betaine primarily takes place in the liver and kidney through the enzyme betaine-homocysteine methyltransferase (BHMT). This enzyme helps transfer the methyl group from betaine to the accumulated intermediate homocysteine. The addition of this methyl group converts homocysteine back into methionine and dimethylglycine (DMG). DMG is then further broken down into sacrosine and glycine (1, 7).

## Transsulfuration Pathway and Cystathionine-β-Synthase

As mentioned above, cystathionine-β-synthase is an enzyme involved in the transsulfuration pathway, which is how the body regulates its levels of sulfur-containing amino acids. The two sulfur-containing amino acids that make up the building blocks of proteins are methionine and cysteine (3, 8). Methionine is an essential amino acid, meaning that it is an amino acid that must be consumed through our diet. Cysteine on the other hand is a conditionally essential amino acid. Under normal physiologic conditions, cysteine can be produced by the body. However, in the presence of diseases such as homocystinuria, cysteine can become an essential amino acid that needs to be ingested through a cysteine supplement. Another example of a sulfur-containing amino acid is homocysteine. Homocysteine is an intermediate in methionine metabolism that, in excess, is responsible for the effects seen in patients with homocystinuria. When methionine is ingested in our diet, it is first converted to S-adenosylmethionine (SAM) via the enzyme methionine adenosyltransferase. SAM then turns into S-adenosylhomocysteine by removal of a methyl (−*CH*_3_) group that is cleaved by a methyl transferase enzyme. The enzyme adenosylhomocysteinase then removes the adenosine group, resulting in the intermediate homocysteine. At this point in the pathway, homocysteine can proceed in one of two directions; it can be converted back into methionine via methionine synthase and its cofactor vitamin *B*_12_ or it can be turned into cystathionine via the enzyme cystathionine-β-synthase and its cofactor vitamin *B*_6_. The cystathionine is then converted to cysteine via cystathionine-γ-lyase. When there is a deficiency or mutation involving CBS, an accumulation of homocysteine and a depletion of cysteine results (1, 3, 9).

## Clinical Manifestations of Cystathionine-β-Synthase Deficiency

Deficiency of CBS results in clinical manifestations in 4 different organ systems within the body as well as psychiatric manifestations. Developmental delay is seen in over half of patients with homocystinuria. This often presents itself in the first few months of life and, if left untreated, continues to worsen as the patient gets older. Studies suggest that over 50% of patients that are left untreated have an IQ below 35 and nearly 90% of untreated patients will have an IQ below 65 (10). A relatively small percentage of untreated patients go on to have average intelligence. Thus, neonatal screening and testing is imperative. If this deficiency is caught at an early age and proper treatment is started, the chance of the child having a lower IQ decreases.

If this condition is left untreated or is poorly managed, psychiatric symptoms often emerge such as hyperactivity, autistic-like ticks and behaviors, obsessive-compulsive behaviors, and personality disorders (11). The nervous system is also often affected in patients with homocystinuria. Neurological signs include seizures, tremors, and hyperactive reflexes along with abnormal electroencephalograph readings. Skeletal symptomologies are also often observed, such as osteoporosis, an increased frequency of vertebral fractures due to lower bone density as well as scoliosis. Patients with these skeletal manifestations can also appear clinically similar to patients with Marfan Syndrome. Marfan syndrome is one of the most commonly inherited disorders involving connective tissue. Marfan syndrome can affect not only the skeletal system but also the cardiac and ocular systems similarly to the situation observed in homocystinuria patients (12). One differing feature between Marfan syndrome patients and homocystinuria patients is that the former often have joint laxity while the later often have more rigid joints. Patients with homocystinuria are also at a higher risk for blood clot formation in both arteries and veins with no regard to vessel size. This risk of blood clot formation also increases the patients' risk for a cerebrovascular attack in young adulthood as well as a pulmonary embolism, both of which are potentially fatal (13). Finally, vision changes often occur in CBS deficient patients. In scenarios where newborn screening was not performed, the first clinical signs seen with this disease are often changes in vision. As patients age, they are at a higher risk for developing astigmatism, cataracts, glaucoma and retinal detachment (14).

## Available Treatments for Cystathionine-β-Synthase Deficiency

The goal in treatment for patients with homocystinuria is to lower homocysteine levels to within normal limits (15). Normal plasma homocysteine is ≤ 15 μM. However, most patients are not started on a treatment regimen until their plasma levels reach 50 μM. One treatment option is the use of a vitamin *B*_6_ supplement. As previously mentioned, vitamin *B*_6_ works as a cofactor for the enzyme CBS. It works by helping CBS catalyze a dehydration reaction where homocysteine is joined with the amino acid serine to produce cystathionine.

**Figure 1:**
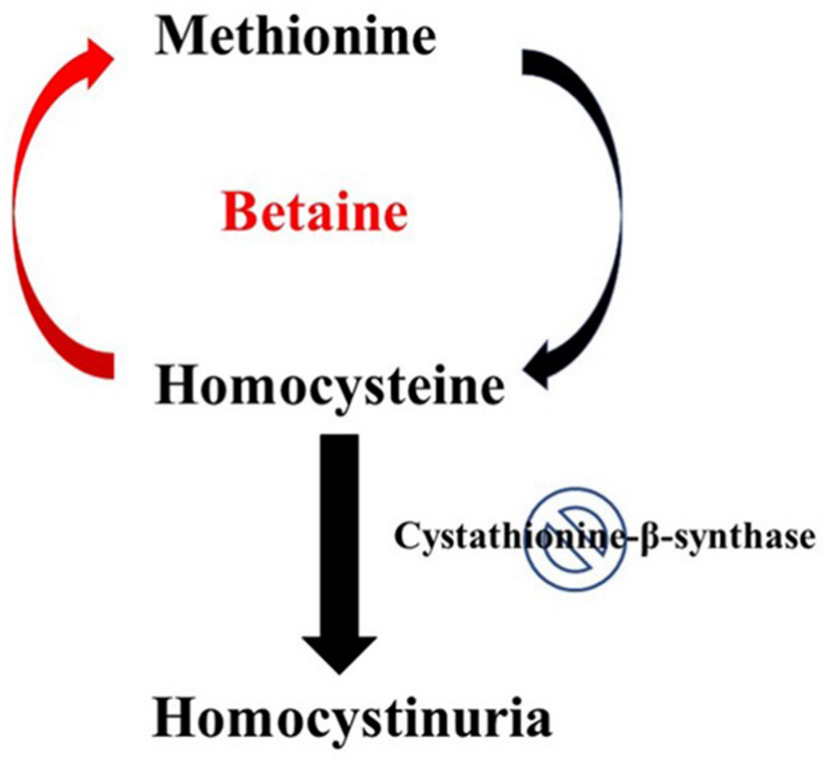
Effect of betaine on Homocystinuria. In healthy individuals, hornocysteine and cysteine can be interconverted in two consecutive steps, catalyzed by cystathionine-p-synthase and cystathionine-y-lyase. The conversion of homocysteine to methionine involves a methylation step in which 5-methyltetrahydrofolate serves as the methyl donor. Classical homocystinuria is caused by the effects of a deficiency (indicated by the stop sign symbol) of the enzyme cystathionine-p-synthase. This deficiency results in the accumulation of homocysteine and a deficiency of cysteine. Intake of betaine by patients with homocystinuria can help resolve this metabolic disorder. Metabolism of betaine primarily takes place through the enzyme betaine-homocysteine methyltransferase. This enzyme helps transfer the methyl group from betaine to the accumulated intermediate homocysteine, thus reducing both homocysteine accumulation and cysteine deficiency.

Patients who can be successfully treated with vitamin *B*_6_ supplementation are referred to as “*B*_6_-responsive” (1, 16). However, this treatment is not successful for all patients. Vitamin *B*_6_ is a cofactor that helps upregulate the enzymatic activity of CBS, but if the deficiency of CBS is severe enough, vitamin *B*_6_ supplementation is not sufficient to adequately regulate homocysteine levels. Another treatment option is for the patient to consume a low-methionine and specialized low- protein diet. Because methionine is an essential amino acid that must be obtained from the diet, minimizing the amount of methionine consumed can lead to reduced stress on the trans-sulfuration pathway. The final treatment option is most suitable for patients who either do not respond to *B*_6_ supplementation or do not wish to follow the recommended restricted diet. This is the use of a vitamin *B*_12_ supplement alongside taking the medication betaine. As mentioned previously, vitamin *B*_12_ works as a cofactor for the enzyme methionine synthase and the drug betaine works as a methyl donor. Together, they can re-methylate homocysteine back to methionine as illustrated in Figure 1 (3). It is important to note however, there have been studies conducted that showed that there was a moderate increase in the overall cholesterol level of patients taking betaine (17). Considering homocystinuria patients are already at risk for cardiovascular complications, this is something that should be closely monitored and taken into consideration while deciding the best treatment option. Lastly, patients with homocystinuria may also experience depletion of certain nutrients depending on which enzyme deficiency is present. For example, patients with the CBS deficiency often require cysteine supplementation while patients with the MTHFR deficiency often require methionine supplementation (1). This is because these enzymes play different roles in methionine metabolism and their deficiency thus has different consequences.

## Betaine Clinical Trials and Dosing

Clinical trials are an important tool for better understanding what therapeutic protocols are best suited for patients with specific diseases. These trials are best done with a large sample size and with specific controls put into place to help researchers ensure that their results are accurate and not due to chance. Homocystinuria is a rare genetic disorder, and therefore performing a large-scale clinical trial on the effectiveness of betaine treatment has proven to be difficult (18). However, smaller scale studies have been conducted on this clinical application of betaine and at what dose it is most effective. One study was performed to examine the effectiveness of betaine in children and adolescents. This study included 6 patients with a known case of homocystinuria due to a cystathionine-β-synthase deficiency. The patients ages ranged from 6 to 17 years old. All patients in this study were all asked to stop their treatment regimen of oral betaine 1 week prior to the study while following a low methionine diet as documented in a food log. Before the initial dose of 100 mg/kg of betaine was given, the patients' blood was drawn, and plasma homocysteine levels were assessed. Upon administering betaine, blood was then drawn from the patients every 2 h for the first 2 h, then every 4 h for 6 h, and finally once at the 24-h mark. These measurements showed that betaine was most effective when given twice daily. The doses that were administered varied between 0 and 1,000 mg/kg/day. Little additional benefit was seen when patients' daily doses exceeded 150 mg/kg/day. Betaine generally is used as a powder that needs to be dissolved in 6–8 ounces of a liquid such as milk, water, or juice. It is recommended that this medication is taken with meals (18, 19). According to the Mayo Clinic, the amount of betaine taken by homocystinuria patients will vary between individuals and will also depend on the dosage prescribed by the physician. In general, the dose regularly used for patients 3 years and older is 1.5 grams taken twice daily with meals. Children that are younger than 3 years old have doses calculated based on their body weight (19). There have been a few studies on the toxicity of betaine when given in large doses. Those done on laboratory rats showed that some adverse effects were noted for a dose of 900 mg/kg/day. It is important to note that these results were not replicated in other studies, so there is a need of further investigation. In 2005 the Panel on Nutrition, Novel Foods and Food Allergens (NDA) investigated adverse effects of betaine reported in human studies. There was one reliable study that was conducted as a placebo-controlled randomized study involving 42 obese patients. These individuals were split into two groups one receiving a placebo and the other receiving a dose of 6 g/kg/day over an 18-week period. There was a noted decrease in plasma homocysteine levels, which is to be expected. However, there was also a decrease seen in the patients' diastolic blood pressure, total serum cholesterol as well as their low-density lipoprotein levels. These types of adverse side effects could have potential serious consequences in patients taking betaine, hence why it is important to follow the dosage and dosing regimen recommended by your physician. In summary, high doses of 6 g/kg/day can result in unwanted effects, while substantially lower doses near 150 mg/kg/day provide clinically beneficial effects for homocystinuria patients (20).

## Benefits of Using Betaine for Cystathionine-β-Synthase Deficiency

The use of betaine by patients with homocystinuria can be very beneficial, especially to those who do not respond to vitamin *B*_6_ or do not wish to adhere to the specialized diet. Understandably, this diet would be difficult to follow considering the patient would be required to cut out most meats and animal produced products, essentially forcing them to follow a strictly vegan type of diet. While this dieting lifestyle may be desirable or feasible for some, it is not realistic to expect all patients to follow said diet. Providing these patients with the option of using betaine allows them more dietary freedom. It is important to note that patients receiving this treatment need to closely monitor their plasma methionine levels with their physician. Levels of methionine >1,000 μM put the patient at risk for brain edema (1).

## Conclusion

Classical homocystinuria is a metabolic disorder that can have detrimental effects on the nervous, ocular, cardiovascular, and skeletal systems and can be controlled by multiple different treatment plans including the use of the drug betaine. Betaine is most beneficial when used by patients who do not respond to vitamin *B*_6_ supplementation and those who do not wish to adhere to the low protein and low methionine diet. Betaine works as a methyl donor with the enzyme betaine-homocysteine methyltransferase and converts homocysteine into methionine. While it is important to monitor methionine levels during this treatment option, it has been successful in treating patients with this disorder.

## Author Contributions

All authors listed have made a substantial, direct and intellectual contribution to the work, and approved it for publication.

## Conflict of Interest

The authors declare that the research was conducted in the absence of any commercial or financial relationships that could be construed as a potential conflict of interest.

## Publisher's Note

All claims expressed in this article are solely those of the authors and do not necessarily represent those of their affiliated organizations, or those of the publisher, the editors and the reviewers. Any product that may be evaluated in this article, or claim that may be made by its manufacturer, is not guaranteed or endorsed by the publisher.
